# Adults’ Adherence to Growth Hormone Replacement in Relation to Medication-Related Beliefs, Coping and Quality of Life - An Exploratory Analysis

**DOI:** 10.3389/fendo.2021.680964

**Published:** 2021-05-24

**Authors:** Sonja Siegel, Nicole Unger, Christine Streetz-van der Werf, Wolfram Karges, Katharina Schilbach, Bernadette Schröder, Janine Szybowicz, Janina Sauerwald, Kathrin Zopf, Agnieszka Grzywotz, Martin Bidlingmaier, Cedric Kirstein, Heide Sommer, Christian J. Strasburger, Ilonka Kreitschmann-Andermahr

**Affiliations:** ^1^ Department of Neurosurgery and Spine Surgery, University of Duisburg-Essen, Essen, Germany; ^2^ Department of Endocrinology, Diabetology and Metabolism, University of Duisburg-Essen, Essen, Germany; ^3^ Division of Endocrinology and Diabetes, Rheinisch-Westfälische Technische Hochschule (RWTH) Aachen University Hospital, Aachen, Germany; ^4^ Medizinische Klinik und Poliklinik IV, Ludwig-Maximilians-Universität (LMU) Klinikum, Munich, Germany; ^5^ Department of Endocrinology, Diabetes and Nutritional Medicine, Charité Universitaetsmedizin, Berlin, Germany; ^6^ Hexal AG, Holzkirchen, Germany

**Keywords:** adult growth hormone deficiency, adherence, quality of life, coping, beliefs about medications

## Abstract

**Introduction:**

Little is known about psychological reasons associated with adherence to growth hormone (GH) replacement therapy (GHRx) in adults. As in other chronic diseases, medication-related beliefs, coping strategies and disease impact on quality of life (QoL) might play an important role. We thus explored these psychological factors in relation to adherence in patients with GH deficiency (GHD) in order to find leverage points for the improvement of adherence.

**Patients and Methods:**

Cross-sectional analysis including 107 adult GHD patients on GHRx who completed self-assessment inventories on health-related QoL (Short-Form SF-36), coping style (Freiburg questionnaire on coping with illness, FKV-LIS) and medication beliefs (Beliefs about Medicine questionnaire, BMQ). Results were correlated to general and GH-specific adherence to medication.

**Results:**

In the BMQ, 92.5% of the patients (n=99) reported a strong belief in the need for their medication, which correlated significantly with general adherence (r_s_ = 0.325). Active coping was significantly related to general (r_s_ = 0.307) and GH-specific adherence (r_s_ = 0.226). Better mental QoL (r_s_ = 0.210) but worse physical QoL (r_s_ = -0.198; all p < 0.05) were related to higher GH-specific adherence. Older age was associated with a higher degree of active coping, a higher belief in the necessity of medication and worse physical QoL.

**Conclusion:**

We provide preliminary data that most GHD patients on GHRx are strongly convinced of their need for medication and that adherence to GHRx is influenced by coping strategies and QoL. Patients with impaired psychological QoL are less able to translate their convictions into good adherence, a phenomenon to be addressed in future research.

## Introduction

Growth hormone (GH) deficiency in adults (aGHD) is associated with an adverse cardiovascular risk and plasma lipid profile, abnormal body composition, reduced bone mass, and impaired quality of life (QoL) (1, 2). These health-related alterations are believed to contribute to the excess morbidity and premature mortality observed in untreated aGHD patients compared to the normal population (3). Growth hormone replacement therapy (GHRx) in adults with recombinant GH, available since the 1990s, is able to ameliorate these negative effects (4–6), however, at the price of long-term treatment, currently involving daily subcutaneous injections. Different from GHRx in children in whom changes in longitudinal growth easily reflect therapy response, the benefits of GHRx in adults may not be as easily perceived. Treatment success in both patient groups is dependent on patients’ continuous therapeutic adherence, i.e. their willingness and ability to follow the recommended injection regime (7).

A number of pediatric studies demonstrate that adherence to GH treatment is suboptimal, with some degree of non-adherence in up to 71% of all pediatric patients and their families as reported in a recent review (8). Preliminary data of Amereller et al. and our group indicate that adherence of aGHD patients to GHRx is higher than in children and adolescents (9, 10). Nevertheless, in clinical practice, a number of aGHD patients decide to refuse recombinant human (rh)GH treatment or to discontinue rhGH therapy over time. Poor therapy adherence as well as discontinuation or refusal of GH treatment may result in reduced QoL and economic burden of those patients (11). Little is known, however, about psychological reasons associated with adherence or non-adherence to GHRx in adults. It can be hypothesized that, as in other chronic diseases, coping strategies, patients’ beliefs about their medication and impact of the disease on the patients’ quality of life might play an important role. Since a better understanding of the psychological influencing factors could provide leverage points for the improvement of adherence, it was the aim of the current analysis to further characterize the beliefs and attitudes leading to adherence to GHRx in patients with GHD. We, therefore, analyzed three major psychological domains outlined below, known to be associated with adherence to medication in other chronic diseases.

### Coping

Coping with a chronic illness includes all of a patient’s cognitive and behavioral efforts to deal with the stress resulting from the disease (12). It is related to adherence to long-term therapies, e.g. hemodialysis in patients with renal disease (13) and medical treatment of type-2 diabetes (14). It has been shown that strategies of coping with chronic diseases differ significantly between patients of different age (15).

### Beliefs About Medications

Beliefs about medications include the patients’ attitudes towards medicines in general and specifically their own prescribed medicaments (16). It has been hypothesized that patients with chronic diseases weigh their beliefs about the necessity of their medication against their concerns about potential adverse effects. Horne and Weimann found this cost-benefit balance to be related to adherence across several chronic illness groups (asthma, renal disease, cardiac disease and oncological diseases) (17).

### Quality of Life

Adherence can also be influenced by the perceived severity of the disease and its impact on day-to-day life (18). In line with this observation, it has been proposed, that health related QoL (hrQOL) and adherence influence each other (19). Patients might decide for the intake of medications based on the perceived impact of the disease on hrQOL and continually reassess hrQOL over the course of the treatment. This reassessment might influence future decisions on adherence, which again might impact hrQOL. Both hrQOL and change in hrQOL over the course of the treatment have been found to be associated with adherence (20, 21).

## Material and Methods

### Study Design

This cross-sectional study was part of an extensive research project on adult GHD and carried out in one large German neurosurgical and four large endocrinological university referral centers during a two-year recruitment period. Adult patients (age between 21 – 80 years) with biochemically proven severe GHD were included. Severe GHD had to be diagnosed either by means of a GH stimulation test performed according to local standards with local cut-offs or by insulin-like growth factor-I (IGF-I) levels more than two standard deviation scores (SDS) below normal in the presence of proven deficiency of three or more other pituitary hormone deficiencies (22). Patients with known active psychotic illnesses and known insufficient fluency of the German language were excluded from participation. A detailed study description and a description of the entire sample of investigated patients have been published previously (10).

For the present research question, a battery of psychological self-rating inventories covering the three domains outlined in the introduction, thought to impact on adherence, was analyzed in the large subgroup of patients on rhGH replacement (n = 107) at the time of the study. The questionnaire results were related to the patients’ adherence to medication in general and to adherence to GHRx, the results of which have been reported previously (10).

The study protocol was approved by the local ethic committees of all participating centers with the lead vote provided by the Ethics Committee of the University of Duisburg-Essen. Patients were included if the signed consent form was returned with the filled-in questionnaires.

### Sample Description

The present analysis includes 107 patients with severe GHD on rhGH treatment. 57 (53.3%) patients were male and 50 (46.7%) were female. The mean age of the study group was 49.9 ± 14.0 years at the time of the study. The etiology of GHD included pituitary adenoma (n = 47), craniopharyngioma (n =11), other tumors of the sellar and suprasellar region (n = 7), congenital (pan)hypopituitarism (n = 9, either genetic or due to hypothalamo-pituitary developmental lesions), empty sella syndrome (n = 6), idiopathic GHD (n = 6), hypophysitis (n = 5), cystic lesions of the pituitary (n = 4), mixed etiologies (such as trauma, Sheehan’s syndrome, sarcoidosis; n = 7). Data on GHD etiology was missing in n = 5 patients. 70 patients (66.7%) had undergone neurosurgery at any time during the disease and 22 patients (21.0%) had received radiation therapy of the pituitary region.

Next to severe somatotropic insufficiency, 85.0% of the study patients suffered from additional gonadotropic insufficiency (n = 91, 79 of them on replacement therapy) and 77.6% had thyrotropic insufficiency (n = 83, all of them substituted). In 73.6% (n = 78 patients) corticotropic insufficiency had been diagnosed. All but one of these patients required regular hydrocortisone replacement. 23.8% (n = 25) of the study patients also suffered from diabetes insipidus which necessitated antidiuretic hormone replacement in all cases. Diabetes mellitus was present in 8.2% (n = 7), hypertension in 38.8% (n = 33) and coronary heart disease in 2.4% of the patients (n = 2). 20.6% of the patients had started rhGH therapy during childhood (n = 21), while 79.4% had started therapy during adulthood (n = 81). Two (1.9%) of all patients did not have a school leaving qualification, 30.2% (n = 32) had a basic school qualification (*Hauptschulabschluss*), 23.6% (n = 25) had a comprehensive school qualification (*Realschulabschlus*s), whereas 44.3% of the patients had a high educational level (*Fachabitur* = university of applied sciences entrance qualification, or higher, n = 47). Sixty-six (62.3%) of the patients were working full-time or part-time at the time of the study.

### Questionnaires

#### Freiburg Questionnaire on Coping With Illness (Freiburger Fragebogen zur Krankheitsverarbeitung; FKV-LIS)

Coping strategies were assessed with the German-language FKV-LIS questionnaire. The FKV-LIS allows the differentiation of a broad spectrum of coping strategies and is widely applied in German-speaking countries to assess illness coping in different disease entities. This 35-item questionnaire is divided into five subscales *(“depressive coping”*, *“active, problem-oriented coping”*; *“distraction and self-affirmation”*, *“religiousness and search for meaning”* and *“trivialization and wishful thinking”*). It assesses coping strategies for dealing with a disease on cognitive, emotional and behavioral levels. While coping strategies cannot be universally categorized as adaptive or maladaptive, *“active and problem-oriented coping”* as well as *“distraction and self-affirmation”* can usually be viewed as more beneficial than *“depressive coping”* and “*trivialization and wishful thinking”*. Answers are based on 5-point Likert scales, with higher scores indicating a higher intensity of coping in a particular domain. Since normative values for coping cannot be established, the raw scores are used for analysis (23). For better readability, we will refer to the scales in the following by abbreviated names, namely “depressive coping”, “active coping”, “distraction”, “religiousness” and “wishful thinking”.

#### Beliefs About Medicine Questionnaire (BMQ)

The BMQ questions cognitive and emotional representations of medication. It has two parts, the BMQ specific and the BMQ General, both of which were used in the present study. The BMQ-General comprises two 4-item factors assessing beliefs that medicines are harmful, addictive poisons which should not be taken continuously (*General Harm*) and that medicines are overprescribed by doctors (*General Overuse*). The BMQ-Specific comprises two 5-item factors assessing beliefs about the necessity of prescribed medication (*Specific Necessity*) and concerns about prescribed medication based on beliefs about the danger of dependency, long-term toxicity and the disruptive effects of medication (*Specific Concerns*) (24). For each of the scales the average score can be calculated. For easier interpretation the results can be dichotomized, with scores above the scale midpoint signifying a strong belief and scores below the scale midpoint signifying a weak belief. To assess the balance between perceived benefits and costs of the prescribed medication, the difference between *Specific Necessity* and *Specific Concerns* can be calculated (17). The resulting score ranges from -20 to 20, with negative values indicating that perceived concerns exceed benefits and positive scores indicating that perceived benefits exceed concerns. In this study, the German translation of the BMQ by U. Opitz was used (25).

#### Short Form-36 (SF-36)

The SF-36 is a generic quality of life outcome measure, consisting of 36 items, which assess health-related QoL of patients during either a one-week (acute) or a four-week (standard) recall period (26). The four-week recall version was used in the present study. The questionnaire has eight domains: *vitality, physical functioning, bodily pain, general health perceptions, physical role functioning, emotional role functioning, social role functioning*, and *mental health*. The answers can be combined to form two global measures, addressing physical (physical component summary score, SF-36 PCS) and mental (mental component summary score, SF-36 MCS) QoL. The raw values are transformed to allow comparison to the general population reference values. The transformed subscales are scaled from 0 to 100; the transformed summary scores have a mean of 50 and a standard deviation (SD) of 10. Higher scores indicate a better QoL.

#### General Adherence Score and GH-Specific Adherence Score

These two questionnaires, developed by the authors have been described in detail previously (10). In brief, the general adherence questionnaire is focused on patients´ adherence with regard to medication in general whereas the GH-specific adherence score focusses on GHRx injections. For both questionnaires, answers could be given on a four-point Likert scale with values ranging between 0 and 3 which were summed up to a general or specific adherence score between 0 and 18, with higher values indicating a higher adherence.

### Data Analysis

Database was generated by Microsoft Access 2010 (Microsoft Office 2010, Microsoft, Redmond/USA). Statistical analyses were conducted using IBM SPSS Statistics 22 (Statistical Package of the Social Sciences, SPSS Inc., Armonk/USA). Descriptive statistics of interval-scaled data were expressed as mean and standard deviations (SD). Categorical data were expressed as absolute frequencies and valid percent (n, %) which means that 100% are the number of available data per parameter out of the 107 included patients. A visual screening of the histogram revealed a severe skewness of the rhGH-specific adherence score and the general adherence score. Therefore, correlation analyses including these scores used the non-parametric Spearman’s Rho coefficient (r_s_). All other correlations were calculated by Pearsons’ correlation coefficient (r) and point-biserial correlation (r_pb_) for dichotomous variables. Where applicable, a p-value of ≤ 0.05 was regarded as statistically significant.

## Results

### Results From the Questionnaires

#### Coping

As indicated by the average score in the FKV-LIS, patients used the strategy of active coping most frequently (3.04 ± 0.86). Distraction (2.82 ± 0.82) and religiousness (2.62 ± 0.75) were less commonly employed. The average scores for depressive coping (2.09 ± 0.81) and wishful thinking (2.09 ± 0.94) were lowest, suggesting that patients used these two coping strategies least.

#### Beliefs About Medicine

In the BMQ, 92.5% of the patients (n = 99) reached a Specific-Necessity Score above the scale midpoint. This documents a strong belief in the need for medication in this patient group. 22.4% of the patients (n = 24) had strong concerns about potential negative effects of their medication. The average Necessity-Concerns Difference was 10.21 ± 5.19, indicating that most patients judged the benefit of their prescribed medication highly, exceeding their concerns about potential negative effects. Only in 4 patients (3.7%), the Necessity-Concerns Difference was negative, meaning that their concerns outweighed the perceived benefit of their medication. The general assumption that medications are overprescribed by doctors was strongly prevalent in 35.5% of the patients (n = 38). Only 5.6% of the patients (n=6) reached a General Harm Score above the scale midpoint, which indicates that the belief that medicines are generally harmful or poisonous was rare.

#### Quality of Life

Physical QoL in the SF-36 (PCS) was reduced to 1 SD below normal in 13.3% (n = 14) and to 2 SD below normal in 6.7% (n = 7) of the patients. In 66.7% (n = 70) physical QoL was normal and 13.3% (n = 14) reported a physical QoL of at least 1 SD above average. This even distribution of values led to a mean PCS of 47.97 ± 10.31 only slightly below the scale midpoint of 50. Mental QoL (MCS) was 1 SD below average in 12.4% (n = 13) and 2 SD below average in 24.8% (n = 26) of the patients. 60.0% (n = 63) reported normal mental QoL and 2.9% (n=3) reported above average mental QoL. Although the mean MCS was 45.43 ± 11.94 and, thus, also close to the scale midpoint, the large percentage of 24.8% of patients with a mean mental QoL 2 SD below average indicates that mental QoL was severely impaired in a large proportion of the investigated patients. Of the individual subscales, *vitality* (50.1 ± 20.84) and *general health perceptions* (57.17 ± 23.10) received the lowest scores.

#### Adherence

The mean general adherence score (16.17 ± 1.7) and the mean rhGH-specific adherence score (15.82 ± 2.01; as reported already in (10) were almost equally high, indicating good adherence to medication in general as well as GHRx.

**Table 1** gives the results of all questionnaires used in the present study.

**Table 1:** Descriptive results of the questionnaires.

	N	M	SD	Min	Max
Adherence					
General Adherence	107	16.17	1.70	9.00	18.00
rhGH-specific Adherence	107	15.82	2.01	9.00	18.00
FKV-LIS^#^					
Depressive coping	105	2.09	0.81	1.00	4.60
Active coping	105	3.04	0.86	1.00	4.80
Distraction	105	2.82	0.82	1.00	4.40
Religiousness	105	2.62	0.75	1.00	4.20
Wishful thinking	104	2.09	0.94	1.00	5.00
BMQ					
Specific Necessity	107	4.36	0.73	1.80	5.00
Specific Concerns	107	2.32	0.85	1.00	4.60
Necessity-Concerns Difference	107	10.21	5.19	-3.00	20.00
General Overuse	107	2.75	0.91	1.00	5.00
General Harm	107	1.79	0.75	1.00	4.00
SF-36					
Physical functioning	107	81.84	21.99	5.00	100.00
Physical role functioning	107	67.52	39.04	0.00	100.00
Bodily pain	107	76.17	26.62	22.00	100.00
General health perception	107	57.17	23.10	5.00	100.00
Vitality	107	50.51	20.84	5.00	95.00
Social functioning	107	77.45	24.91	12.50	100.00
Emotional role functioning	107	69.81	40.51	0.00	100.00
Mental well-being	107	67.73	19.68	0.00	100.00
Physical Component Score	106	47.97	10.31	16.51	68.54
Mental Component Score	106	45.43	11.94	11.86	60.63

^#^Please note that abbreviated scale names for the FKV-LIS are reported here for better readability. For the full scale names please refer to the Methods section.

### Correlations Between Questionnaire Scores and Adherence

#### Coping

The only coping strategy significantly related to adherence was active coping. It correlated positively with the General Adherence and GH-specific Adherence Scores of our self-developed scale. This indicates that the more patients relied on active coping, the better was their adherence to medication in general and to GHRx. All other coping strategies were unrelated to General Adherence and GH-specific Adherence.

#### Beliefs About Medicine

**Figure 1:**
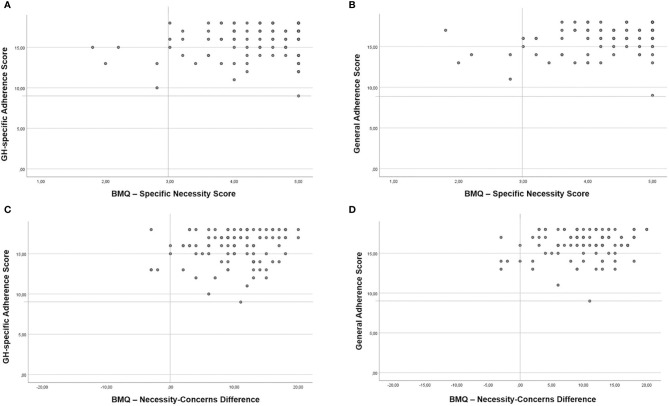
BMQ Specific Necessity Scores in relation to GH-specific Adherence **(A)** and General Adherence **(B)** and BMQ Necessity-Concerns Difference in relation to GH-specific Adherence **(C)** and General Adherence **(D)**.

**Figure 1** illustrates the limited variability of the BMQ *Specific-Necessity* scores and general and GH-specific adherence scores, indicating strong beliefs and high adherence to medication and GHRx in the great majority of the investigated patients. The subscale *Specific-Necessity* thus only correlated moderately with General Adherence, but not with GH-specific adherence. For the same reason, the Necessity-Concerns Difference, which measures the balance between perceived benefits and costs of the prescribed medication, was not correlated with adherence (also see **Figure 1**).

The *General Harm* subscale of the BMQ, was negatively correlated to GH-specific adherence. Thus, patients who had the opinion that medicines are addictive or poisonous and should not be taken continuously were more likely to be inadherent.

#### Quality of Life

**Figure 2:**
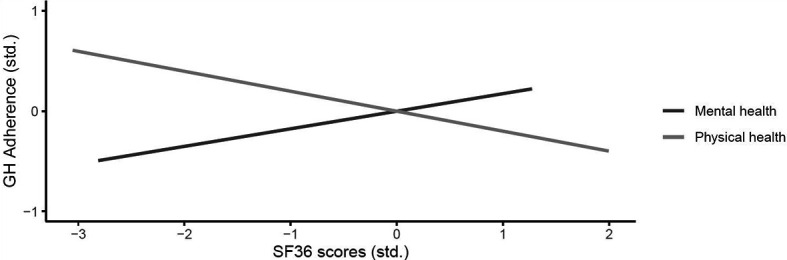
Correlation between GH-specific adherence and the SF-36 sum scores (z-standardized).

General adherence was significantly negatively related to mental QoL as measured by the SF-36 MCS. Thus, the worse the mental QoL was, the lower was the General Adherence Score. The SF-36 subscales Vitality and Mental well-being correlated positively with General Adherence, as well. GH-specific Adherence was also significantly correlated with SF-36 PCS and MCS. Interestingly, better mental QoL but worse physical QoL were related to higher adherence (cf. **Figure 2**). None of the SF-36 subscales yielded significant correlations with GH-specific adherence.

**Table 2** gives the nonparametric correlations between the adherence scores and the questionnaire results.

**Table 2:** Nonparametric correlations between general and rhGH-specific adherence and the results from the psychometric questionnaires.

		General Adherence		Adherence GH	
		r_s_	p	r_s_	p
Adherence					
rhGH specific Adherence	107	0.564^***^	< 0.001	–	–
FKV-LIS^#^					
Depressive coping	105	-0.128	0.193	-0.132	0.179
Active coping	105	0.307^**^	0.001	0.226^*^	0.020
Distraction	105	0.188	0.054	0.182	0.063
Religiousness	105	0.046	0.640	-0.015	0.877
Wishful thinking	104	-0.083	0.405	0.003	0.974
BMQ					
Specific Necessity	107	0.325^**^	0.001	0.145	0.135
Specific Concerns	107	0.108	0.268	-0.046	0.639
Necessity-Concerns Difference	107	0.142	0.145	0.104	0.286
General Overuse	107	0.004	0.969	0.036	0.716
General Harm	107	-0.177	0.068	-0.193^*^	0.046
SF-36					
Physical functioning	107	-0.019	0.848	-0.074	0.451
Physical role functioning	107	-0.068	0.488	-0.110	0.260
Bodily pain	107	0.005	0.957	-0.079	0.417
General health perception	107	-0.080	0.414	-0.101	0.299
Vitality	107	0.225^*^	0.020	0.173	0.074
Social functioning	107	0.156	0.108	0.132	0.176
Emotional role functioning	106	0.144	0.141	0.044	0.656
Mental well-being	107	0.252^**^	0.009	0.137	0.159
Physical Component Score	106	-0.170	0.082	-0.198^*^	0.042
Mental Component Score	106	0.255^**^	0.008	0.210^*^	0.030

^#^Please note that abbreviated scale names for the FKV-LIS are reported here for better readability. For the full scale names please refer to the Methods section.*p< 0.05, **p< 0.01, ***p < 0.001.

### Correlations Between Coping and Quality of Life

The SF-36 MCS was negatively correlated with depressive coping (r = -0.705, p < 0.001), wishful thinking (r = -0.459, p < 0.001) and religiousness (r = -0.257, p = 0.008). Patients, who engaged more frequently in these coping strategies, thus, had a lower psychological QoL. The SF-36 PCS was only correlated to religiousness (r = -0.262, p = 0.007), indicating that the physical QoL was worse in those patients who stated to rely on religiousness to cope with their illness.

### Associations With Age

Older age was significantly related to presence of comorbidities (r_pb_ = 0.221, p = 0.022) and number of additional hormone deficiencies (r = 0.236, p = 0.015). Accordingly, age correlated negatively with the SF-36 PCS (r = -0.228, p = 0.019), signifying a lower physical QoL in older patients. Since the standardized SF-36 sum scores takes gender and age specific norm values into account, the reported significant correlation between age and lower PCS scores in the present study thus exceeds the normal physical decline of older patients in a healthy population. The SF-36 MCS tended to be higher than in younger patients, albeit not significantly so (r = 0.168, p = 0.086). Older age was associated with a higher degree of active coping (r = 0.227, p = 0.020) and distraction (r = 0.195, p = 0.047), but not with religiousness. Older patients also stated to believe in the necessity of their medication more often than younger patients (BMQ Specific Necessity, r = 0.206, p = 0.033). On the other hand, the general belief that medications were overprescribed was more frequently shared by older patients (r = 0.235, p = 0.015). Their specific concerns and belief in the general harm of medications did not differ from younger patients, neither did their Necessity-Concerns Difference (all r<0.170, ns.).

## Discussion

### Characterization of the Study Group

In the present study we investigated for the first time the association between psychological factors and adherence to medication in general and to GH replacement in adult patients with hypopituitarism including GHD. We found our investigated patients to rely on adaptive coping strategies such as active coping and self-distraction most often and to be strongly convinced of the necessity of their medication. Physical QoL of the entire sample was evenly distributed around the normative mean. However, older patients reported a significantly worse physical QoL than the younger ones, exceeding the normal decline of physical QoL in the general population, which might be explained by the high prevalence of comorbidities and additional hormone deficiencies in this subgroup. Mental QoL, on the other hand, was severely impaired with 2 SD below average in almost a quarter of our study population and unrelated to age. While a number of studies demonstrate that adults with untreated GHD have impaired QoL as compared to the general population (2, 27), there are only scarce data on the QoL of patients on stable, long-term GHRx, stemming from only one investigation published in 2003 (28). Despite being on stable GHRx for three years and adequate replacement of other hormones, multiple aspects of QoL remained impaired in comparison to age- and sex-matched controls in the then investigated participants. However, despite the relatively high percentage of patients with impaired mental well-being in our group, a comparison of the individual subscales of the SF-36 between the historic cohort mentioned above [view table 3 in their original publication (28)] and the present one indicates a now overall better QoL of patients with hypopituitarism on GHRx, which is perhaps related to more routine and confidence with hormonal replacement regimes evolving over time.

### Influence of Psychological Factors and Age on Adherence

Of the psychological constructs investigated, we found the coping strategy of active coping to be most associated with adherence to GHRx and general adherence, followed by the negative influence of poor mental QoL on both adherence scales. Worse physical QoL was significantly associated with a better adherence to GHRx and displayed an insignificant trend for a better adherence to medication in general. Expectedly, the subscale of the BMQ exploring the belief that medications are addictive poisons was associated with significantly worse adherence to GHRx and, again, showed an insignificant trend to worse adherence to medication in general. Older patients were significantly more likely to use active coping and were more strongly convinced of the need for their medication, despite their more frequent belief that medications are generally overprescribed.

The association of better adherence and predominant use of beneficial coping strategies such as active coping in our study did not come unexpected and fits well into the literature on adherence in other chronic diseases such as rheumatoid arthritis and diabetes mellitus (29, 30). The result now also provides a psychological explanation for our previous finding that adherence to GHRx is age-dependent (10) in that older patients in our subanalysis succeeded in coping more effectively with their illness than the younger ones. Moreover, their strong conviction of the necessity of medication might be fueled by their disproportionally poor physical QoL, resulting in a better adherence to GHRx. One could have expected the same to apply to the relationship between mental QoL and adherence, in the sense that more severely impaired mental QoL would be a trigger for higher adherence in order to ameliorate this impairment. In the present study, however, we found the opposite effect - despite a high belief in the necessity for the taken medication, not only in older patients but the entire study group. How can this apparent contradiction be resolved? Acknowledging the high concordance between depression and impaired mental QoL in general, as well as the association between impaired mental QoL and depressive coping in the investigated study group, we assume a high prevalence of depression in our patients and propose the following explanation: Strong evidence suggests, that depression is associated with a poorer adherence to treatment in many physical diseases (31). It has further been proposed, that this relation is mediated by psychological processes, as, for example, proposed in the theory of planned behavior (TPB) (32). In short, this model states that favorable behavioral intentions (i.e., the intent to adhere to GHRx) can only then be translated into actual behavior (i.e., good adherence), if the individual has control over his/her behavioral engagement. Since depression has consistently been related to negative illness beliefs, helplessness and lack of perceived illness control [for an overview see (33)], it may be concluded that patients with severely impaired mental QoL are less able to realize their intent to adhere to GHRx and other medications because of a lack of behavioral control over their illness and adequate coping strategies. A similar relationship has already been shown in breast cancer survivors (34), strengthening our hypothesis.

### Strengths, Limitations and Implications for Further Research

To our knowledge, the present study is the first to explore psychological influencing factors of adherence to GHRx in adults with GHD. The large dataset of this cohort provided us the opportunity for a stable statistical analysis, taking into account three major psychological domains associated with adherence to medication in other diseases. However, the study was exploratory in nature. While it provides first indications that coping strategies, beliefs about medication and QoL are associated with adherence to GHRx, the results await further confirmation using a study design with independent hierarchical regression analysis, in which the order of entry is assigned by the researcher. Also, we did not specifically investigate depression as a comorbidity and its potential impact on adherence to GHRx. Last, but not least, we acknowledge a potential response bias in that we cannot rule out that predominantly highly motivated and adherent patients participated in this study, who might not constitute a representative sample of the general group of patients on GHRx. However, this is a problem shared by all studies relying on self-reporting measures and cannot be avoided in patient-reported outcome research.

Our results open avenues for further research, especially pertaining to the theory of planned behavior and its relationship to depression. Future studies should specifically address behavioral control and the intention to adhere to GHRx, especially in younger adults and patients in the transition period who might not have learned to cope effectively with chronic illness yet. For clinical practice, it would be interesting to learn if GHRx with long-acting GH preparations could facilitate adherence in those patients with poor psychological quality of life and lack of effective coping strategies. Moreover, the influence of interventions targeted at improving coping strategies in patients on GHRx on adherence needs to be investigated.

## Conclusion

These pilot study data indicate that most GHD patients on GHRx are strongly convinced of the need for their medication and that adherence to GHRx is influenced by coping strategies and QoL. Patients with impaired mental QoL are less able to translate their convictions into good adherence. We believe this phenomenon to be caused by a lack of behavioral illness control and negative coping strategies, a hypothesis to be investigated in future research. Since younger patients coped less effectively and had a poorer adherence to GHRx, interventions to improve adherence should be developed for and investigated especially in this patient group.

## Data Availability Statement

The raw data supporting the conclusions of this article will be made available by the authors, without undue reservation.

## Ethics Statement

The studies involving human participants were reviewed and approved by the Ethics Committee of the Medical Faculty of the University of Duisburg-Essen. The patients/participants provided their written informed consent to participate in this study.

## Author Contributions

SS, NU, CS-W, WK, KS, MB, CS and IK-A contributed to the conception of the study. BS, JSz, JSa, KZ and AG collected the data, SS, IK-A and CK conducted the statistical analysis. All authors took part in the interpretation of the data. SS, CS and IK-A wrote the first draft of the paper. All authors contributed to the article and approved the submitted version.

## Conflict of Interest

MB, CS and IK-A are members of the German PATRO Board and, as such, have received consulting and speaker fees as well as travel support from Hexal AG. WK has received honoraria from NovoNordisk and Novartis. CS-W has received speakers’ fees and/or travel grants from NovoNordisk and Pfizer. SS and AG have received travel grants from Hexal AG.

The study was supported by an independent-investigator initiated grant from Hexal Germany. The funder had no role in the study design, data collection, data analysis or decision to publish. However, the expertise of HS, who is an employee of Hexal with a long-standing scientific background in human growth hormone deficiency, was appreciated for the interpretation of the data and manuscript revision. She, therefore, is a co-author of the manuscript.

The remaining authors declare that the research was conducted in the absence of any commercial or financial relationships that could be construed as a potential conflict of interest.

## References

[1] Melmed S. Pathogenesis and Diagnosis of Growth Hormone Deficiency in Adults. N Engl J Med (2019) 380:2551–62. 10.1056/NEJMra1817346 31242363

[2] McGauley G Cuneo R Salomon F Sönksen PH. Growth Hormone Deficiency and Quality of Life. Horm Res (1996) 45:34–7. 10.1159/000184756 8742116

[3] Erfurth EM. Update in Mortality in GH-Treated Patients. J Clin Endocrinol Metab (2013) 98:4219–26. 10.1210/jc.2013-2415 24030944

[4] Maison P Griffin S Nicoue-Beglah M Haddad N Balkau B Chanson P. Impact of Growth Hormone (GH) Treatment on Cardiovascular Risk Factors in GH-Deficient Adults: A Metaanalysis of Blinded, Randomized, Placebo-Controlled Trials. J Clin Endocrinol Metab (2004) 89:2192–9. 10.1210/jc.2003-030840 15126541

[5] Crespo I Santos A Webb SM. Quality of Life in Patients With Hypopituitarism. Curr Opin Endocrinol Diabetes Obes (2015) 22:306–12. 10.1097/MED.0000000000000169 26103454

[6] Mo D Blum WF Rosilio M Webb SM Qi R Strasburger CJ. Ten-Year Change in Quality of Life in Adults on Growth Hormone Replacement for Growth Hormone Deficiency: An Analysis of the Hypopituitary Control and Complications Study. J Clin Endocrinol Metab (2014) 99:4581–8. 10.1210/jc.2014-2892 25233155

[7] World Health Organization. Adherence to Long-Term Therapies: Evidence for Action. Geneva, Switzerland: WHO (2003). Available at: https://www.who.int/chp/knowledge/publications/adherence_introduction.pdf.

[8] Graham S Weinman J Auyeung V. Identifying Potentially Modifiable Factors Associated With Treatment Non-Adherence in Paediatric Growth Hormone Deficiency: A Systematic Review. Horm Res Paediatr (2018) 90:221–7. 10.1159/000493211 30522126

[9] Amereller F Schilbach K Schopohl J Störmann S. Adherence, Attitudes and Beliefs of Growth Hormone Deficient Patients - a Questionnaire-Based Cohort Study. Exp Clin Endocrinol Diabetes (2021) 129:112–7. 10.1055/a-0956-1919 31266067

[10] Kreitschmann-Andermahr I Siegel S Unger N Streetz-van der Werf C Karges W Schilbach K. Motivation for and Adherence to Growth Hormone Replacement Therapy in Adults With Hypopituitarism: The Patients’ Perspective. Pituitary (2020) 23:479–87. 10.1007/s11102-020-01046-y PMC7426293 32441023

[11] Loftus J Camacho-Hubner C Hey-Hadavi J Goodrich K. Targeted Literature Review of the Humanistic and Economic Burden of Adult Growth Hormone Deficiency. Curr Med Res Opin (2019) 35:963–73. 10.1080/03007995.2018.1546682 30411985

[12] Ridder DD Schreurs K. Coping, Social Support and Chronic Disease: A Research Agenda. Psychol Health Med (1996) 1:71–82. 10.1080/13548509608400007

[13] Christensen AJ Benotsch EG Wiebe JS Lawton WJ. Coping With Treatment-Related Stress: Effects on Patient Adherence in Hemodialysis. J Consult Clin Psychol (1995) 63:454. 10.1037/0022-006X.63.3.454 7608358

[14] Smalls BL Walker RJ Hernandez-Tejada MA Campbell JA Davis KS Egede LE. Associations Between Coping, Diabetes Knowledge, Medication Adherence and Self-Care Behaviors in Adults With Type 2 Diabetes. Gen Hosp Psychiatry (2012) 34:385–9. 10.1016/j.genhosppsych.2012.03.018 PMC3383912 22554428

[15] Felton BJ Revenson TA. Age Differences in Coping With Chronic Illness. Psychol Aging (1987) 2:164. 10.1037/0882-7974.2.2.164 3268205

[16] Horne R. Patients’ Beliefs About Treatment: The Hidden Determinant of Treatment Outcome? J Psychosom Res (1999) 47(6):491–5. 10.1016/S0022-3999(99)00058-6 10661596

[17] Horne R Weinman J. Patients’ Beliefs About Prescribed Medicines and Their Role in Adherence to Treatment in Chronic Physical Illness. J Psychosom Res (1999) 47(6):491–5. 10.1016/S0022-3999(99)00057-4 10661603

[18] DiMatteo MR Haskard KB Williams SL. Health Beliefs, Disease Severity, and Patient Adherence: A Meta-Analysis. Med Care (2007) 45(6):521–8. 10.1097/MLR.0b013e318032937e 17515779

[19] Côté I Farris K Feeny D. Is Adherence to Drug Treatment Correlated With Health-Related Quality of Life? Qual Life Res (2003) 12:621–33. 10.1023/A:1025180524614 14516172

[20] Holt EW Muntner P Joyce CJ Webber L Krousel-Wood MA. Health-Related Quality of Life and Antihypertensive Medication Adherence Among Older Adults. Age Ageing (2010) 39:481–7. 10.1093/ageing/afq040 PMC2886202 20513770

[21] Bernstein D Kleinman L Barker CM Revicki DA Green J. Relationship of Health-Related Quality of Life to Treatment Adherence and Sustained Response in Chronic Hepatitis C Patients. Hepatology (2002) 35:704–8. 10.1053/jhep.2002.31311 11870387

[22] Hartman ML Crowe BJ Biller BM Ho KK Clemmons DR Chipman JJ. Which Patients Do Not Require a GH Stimulation Test for the Diagnosis of Adult GH Deficiency? J Clin Endocrinol Metab (2002) 87:477–85. 10.1210/jcem.87.2.8216 11836272

[23] Muthny FA. Freiburger Fragebogen Zur Krankheitsverarbeitung. Fkv. Beltz, Weinheim, Germany (1989).

[24] Horne R Weinman J Hankins M. The Beliefs About Medicines Questionnaire: The Development and Evaluation of a New Method for Assessing the Cognitive Representation of Medication. Psychol Health (1999) 14:1–24. 10.1080/08870449908407311

[25] Opitz U. Subjektive Krankheits- Und Behandlungskonzepte Bei Patientinnen Mit Fibromyalgiesyndrom. Freiburg, Germany: FreiDok plus Universitätsbibliothek Freiburg (2011). Available at: https://freidok.uni-freiburg.de/data/8032.

[26] Bullinger M. Erfassung der gesundheitsbezogenen Lebensqualität mit dem SF-36 Health Survey [Assessment Of Health Related Quality Of Life With The SF-36 Health Survey]. Rehabilitation (Stuttg) (1996) 35:XVII–XXVII; quiz XXVII. 8975342

[27] Badia X Lucas A Sanmartí A Roset M Ulied A. One-Year Follow-Up of Quality of Life in Adults With Untreated Growth Hormone Deficiency. Clin Endocrinol (Oxf) (1998) 49:765–71. 10.1046/j.1365-2265.1998.00634.x 10209564

[28] Malik IA Foy P Wallymahmed M Wilding JP MacFarlane IA. Assessment of Quality of Life in Adults Receiving Long-Term Growth Hormone Replacement Compared to Control Subjects. Clin Endocrinol (Oxf) (2003) 59:75–81. 10.1046/j.1365-2265.2003.01799.x 12807507

[29] Berner C Erlacher L Fenzl KH Dorner TE. Medication Adherence and Coping Strategies in Patients With Rheumatoid Arthritis: A Cross-Sectional Study. Int J Rheumatol (2019) 2019:4709645. 10.1155/2019/4709645 30949207 PMC6425297

[30] Gonzalez JS Tanenbaum ML Commissariat PV. Psychosocial Factors in Medication Adherence and Diabetes Self-Management: Implications for Research and Practice. Am Psychol (2016) 71:539–51. 10.1037/a0040388 PMC5792162 27690483

[31] Raynor D Wing R Phelan S. Depression and Adherence to Medical Advice. Depression and Physical Illness. Cambridge University Press (2006). p. 369–96. 10.1017/CBO9780511544293.018

[32] Ajzen I. The Theory of Planned Behavior. Organ Behav Hum Decis Process (1991) 50:179–211. 10.1016/0749-5978(91)90020-T

[33] Capobianco L Faija C Husain Z Wells A. Metacognitive Beliefs and Their Relationship With Anxiety and Depression in Physical Illnesses: A Systematic Review. PloS One (2020) 15:e0238457. 10.1371/journal.pone.0238457 32911486 PMC7500039

[34] Manning M Bettencourt BA. Depression and Medication Adherence Among Breast Cancer Survivors: Bridging the Gap With the Theory of Planned Behaviour. Psychol Health (2011) 26:1173–87. 10.1080/08870446.2010.542815 21929477

